# Impact of a paediatric-adult care transition programme on the health status of patients with sickle cell disease: study protocol for a randomised controlled trial (the DREPADO trial)

**DOI:** 10.1186/s13063-019-4009-9

**Published:** 2020-02-10

**Authors:** Delphine Hoegy, Nathalie Bleyzac, Alexandra Gauthier-Vasserot, Giovanna Cannas, Angélique Denis, Arnaud Hot, Yves Bertrand, Pauline Occelli, Sandrine Touzet, Claude Dussart, Audrey Janoly-Dumenil, DREPADO study group, C. Pivot, C. Pondarre, F. Galactéros, E. Fois, M. De Montalembert, J. B. Arlet, G. Elana, K. Michaux, C. Guitton, C. Chantalat, S. Eyssette-Guerreau, L. Blum

**Affiliations:** 1 EA 4129 P2S Parcours Santé Systémique-Université Claude Bernard Lyon 1, Université Lyon 1, Lyon, France; 2 Pharmacie Hôpital Edouard Herriot, Hospices Civils de Lyon, 5 place d’Arsonval, 69003 Lyon, France; 3 Institut des Sciences Pharmaceutiques et Biologiques, Université Claude Bernard Lyon 1, Université Lyon 1, Lyon, France; 4 INSERM U1111-CNRS UMR 5308 ENS Lyon, Université Lyon 1, Lyon, France; 5 EMR 3738, PK/PD modeling in oncology, Université Claude Bernard Lyon 1, Université Lyon 1, Lyon, France; 6 Centre Antipoison, Hospices Civils de Lyon, Lyon, France; 7 Institut d’Hématologie et d’Oncologie Pédiatrique de Lyon, Hospices Civils de Lyon, Lyon, France; 8 Laboratoire Interuniversitaire de Biologie de la Motricité (LIBM) EA7424, Université Claude Bernard Lyon 1, Université Lyon 1, Lyon, France; 9 Laboratoire d’Excellence du Globule Rouge (Labex GR-Ex), PRES Sorbonne, Paris, France; 10 Médecine Interne, Hôpital Edouard Herriot, Hospices Civils de Lyon, Lyon, France; 11 Pôle Santé Publique, Hospices Civils de Lyon, Lyon, France; 12 Centre de Référence Constitutif Syndromes Drépanocytaires Majeurs, Thalassémies et Autres Pathologies Rares du Globule Rouge et de l’Erythropoïèse, Hospices Civils de Lyon, Lyon, France; 13 EA 7425 HESPER Health Services and Performance Research, Université Lyon 1, Lyon, France; 14 Pharmacie Centrale, Hospices Civils de Lyon, Lyon, France

**Keywords:** Sickle cell disease, Paediatric-adult care transition

## Abstract

**Background:**

Thanks to advancements in medical care, a majority of patients with sickle cell disease (SCD) worldwide live beyond 18 years of age, and therefore, patients initially followed in paediatric departments are then transferred to adult departments. This paediatric-adult care transition is a period with an increased risk of discontinuity of care and subsequent morbidity and mortality. During this period, the patient will have to manage new interlocutors and places of care, and personal issues related to the period of adolescence. To take into consideration all these aspects, an interesting approach is to use the whole system approach to the patient, as presented in the biopsychosocial approach. The aim of this trial is to evaluate the impact of the proposed biopsychosocial paediatric-adult transition programme.

**Methods:**

The DREPADO study is a multicentre randomised control trial comparing a control group (Arm A) to an interventional group with a paediatric-adult transition programme based on a biopsychosocial approach (Arm B). To be included, patients should have the SS, SC, or Sβ form of sickle cell disease and be aged between 16 and 17 years. The randomisation in a 1:1 ratio assigns to Arm A or B. The primary outcome is the number of hospital admissions and emergencies for complications in the index hospital, in the 2 years after the first consultation in the adult department of care. Secondary outcomes consider the quality of life, but also include coping skills such as sense of self-efficacy and disease knowledge. To provide patient and parent knowledge and coping skills, the transition programme is composed of three axes: educational, psychological, and social, conducted individually and in groups.

**Discussion:**

By providing self-care knowledge and coping skills related to SCD and therapeutics, helping empower patientsin relation to pain management and emotions, and facilitating the relationship to oneself, others, and care in Arm B of the DREPADO study, we believe that the morbidity and mortality of patients with SCD may be reduced after the proposed paediatric-adult transition programme.

**Trial registration:**

ClinicalTrials.gov, ID: NCT03786549; registered on 17 December 2018; https://clinicaltrials.gov/.

## Contributions to the literature

- Sickle cell disease (SCD) is poorly represented in the literature on chronic diseases, in particular on the management of care.
- During the paediatric-adult transition, there is an increased risk of discontinuity of care and subsequent morbidity and mortality, so improvement is essential. Based on scientific evidence, the proposed paediatric-adult transition programme used a biopsychosocial approach to consider the patient and their family within the whole system.
- Using the methodology of randomised controlled trials, this protocol contributes to the construction of evidence on the paediatric-adult transition of patients with SCD.

## Background

Sickle cell disease (SCD) is a chronic, genetic disease, widespread both around the world and in France. After diagnosis, patients with SCD require medical follow-ups, which are essential to prevent complications. The most frequent complications are vaso-occlusive crises (VOC; which are painful), acute chest syndrome (ACS), and stroke. Thanks to advancements in medical care, a majority of SCD patients worldwide live beyond 18 years of age [1], and therefore, patients initially followed in paediatric departments are then transferred to adult departments.

For patients with chronic disease such as SCD, this paediatric-adult care transition is a period with an increased risk of discontinuity of care [2, 3]. During this period, adherence to medication is sub-optimal [1, 4] and the rate of missed clinical appointments is high [5]. Furthermore, the highest morbidity and mortality is observed between 17 and 18 years of age [6], and young adults frequently use acute care (emergency care and hospitalisations) after paediatric-adult transition [7].

During the paediatric-adult transition, the patient will have to manage new interlocutors and places of care, as well as a different organisation of this. An important aspect to improve the health status of patients is, therefore, to involve both paediatric and adult departments of care in the transition [3]. In addition, the patient will have to manage personal issues related to the period of adolescence [8]: a period of psychological upheaval and adaptation of family roles [9]. To assess the needs of patients with SCD during this transition period, a qualitative study was conducted in Lyon (France). Parents, as well as adolescent and adult patients with SCD, were individually interviewed about it. Results revealed individual and also family needs that concerned knowledge of the disease, coping skills, therapeutics, and psychosocial aspects [10].

To take into consideration all these aspects, an interesting approach is to regard the patient with a whole system approach, as presented in the biopsychosocial or social-ecological model [11]. Based on this approach, the Social-Ecological Model of Adolescent and Young Adult Readiness to Transition (SMART) proposes a conceptual framework that describes factors influencing paediatric-adult transition in SCD [12]. This includes bioclinical factors such as complications, psychological factors such as pain perception, and social factors such as scholar absenteeism [12]. Some of these factors may be modified and therefore these could become part of the patient’s coping skills [11, 12]. For instance, a coping skill for managing complications may be knowing when to go to the hospital based on a patient’s own pain threshold. For this, a pluridisciplinary team is required [12].

Currently, only pilot studies of transition programmes based on biopsychosocial approaches have been performed [13–16], with encouraging primary results [13].

The DREPADO open-label, individual, multicentre, randomised controlled trial (RCT) proposes to assess a paediatric-adult transition program for patients with SCD based on a biopsychosocial approach and SMART compared to standard care management. As compared to available studies that have investigated the paediatric-adult care transition [13], the originality of this study is the biopsychosocial approach coupled to its randomised controlled design. The purpose of the article is to present the DREPADO study protocol.

## Methods/Design

### The aim, design, and setting of the study

The DREPADO RCT seeks to assess the impact of a paediatric-adult transition programme. This trial is an interventional study. It is an open-label, individual, multicentre RCT comparing a control group (Arm A) to an interventional group with a paediatric-adult transition SCD programme based on the biopsychosocial approach (Arm B).

The DREPADO trial is a French national RCT including centres specialised in SCD patient care, involved in the SCD health network (*Maladie Chronique du Globule Rouge et des autres maladies de l’érythropoïèse* Filière - MCGRE) accredited by the French Ministry of Health and motivated to participate in this study. The coordinating centre is Lyon University Hospital, and five other centres will participate (Créteil, APHP Kremlin-Bicêtre, Martinique, APHP Necker, Pontoise).

The follow-up period is a maximum of 4 years for each patient. Outcomes is assessed at baseline (T-inclusion), at transfer date – which corresponds to the first consultation in the adult department of care (T-transfer), 12 months after transfer (T-12), and 24 months after transfer (T-24).

### Study subjects

The study focuses on adolescent patients with SCD and their caregivers. The patient-caregiver dyad is included. The inclusion period is expected to be 2 years.

#### Inclusion criteria

Patients are included if they fulfil the following conditions: having the SS, SC, or Sβ form of SCD; aged between 16 and 17 years; orally agreeing to participate. The caregiver is included if they fulfil the following conditions: parent or legal guardian of the patient; aged more than 18 years; having provided a signed written consent for him/herself and for the patient.

#### Non-inclusion criteria

Patients and caregivers with any of the following conditions are excluded: presence of any known and major cognitive or psychiatric disorder that could, according to the judgement of the investigator, hamper intervention or evaluation, and/or familial history of such disorder; patients considered healed after stem cell transplantation. No concomitant care, interventions, or enrolment to other trials are prohibited by participation in the DREPADO trial.

#### Withdrawal and discontinuation

Participants can withdraw voluntarily at any time during the trial. Participants who are not present at intervention and/or evaluation are contacted by telephone; after four unsuccessful calls, participants are considered dropouts.

### Sample size and recruitment

A total of 196 (98 in each group) is required to detect at least a 15% reduction of the hospitalisation rate for complications 2 years after the transfer in the intervention group compared to the control group. This reduction assumes a hospitalisation rate of 3.5 per patient per year [7] with 80% power and the use of a two-sided test at a significance level of 5% and a 10% inflation factor to anticipate study deviations (missing data, withdrawal, loss to follow-up).

Participants are recruited in the hospital when they come for a medical consultation or at the end of a hospitalisation for complications. Potential participants are screened. The investigator informs the patient and caregiver about the study, following which the patient gives oral consent and the patient’s caregiver the written informed consent for patient participation. The information note and consent form is presented in Additional file 1. When the patient legally becomes an adult (i.e. at 18 years of age in France), he will give his/her own written informed consent.

### Randomisation and blinding

Participants are randomly assigned to one of the two groups at a 1:1 ratio using a minimisation algorithm. The minimisation factors are the centre, the type of SCD, and the occurrence of VOC during the year before inclusion. Allocation is done using a central randomisation system (Ennov Clinical, version 7.5.710.4, San Francisco, CA, USA). At each centre, a clinical research associate delegated by the investigator and who is trained in good clinical practice (GCP) logs into the central randomisation system and inputs patient information; a random number and group assignment are then immediately given by the system. Because DREPADO is an open-label trial, the clinical research associate directly informs the patient-caregiver dyad of the arm to which they are assigned (Arm A or B).

### Study scheme

**Fig. 1:**
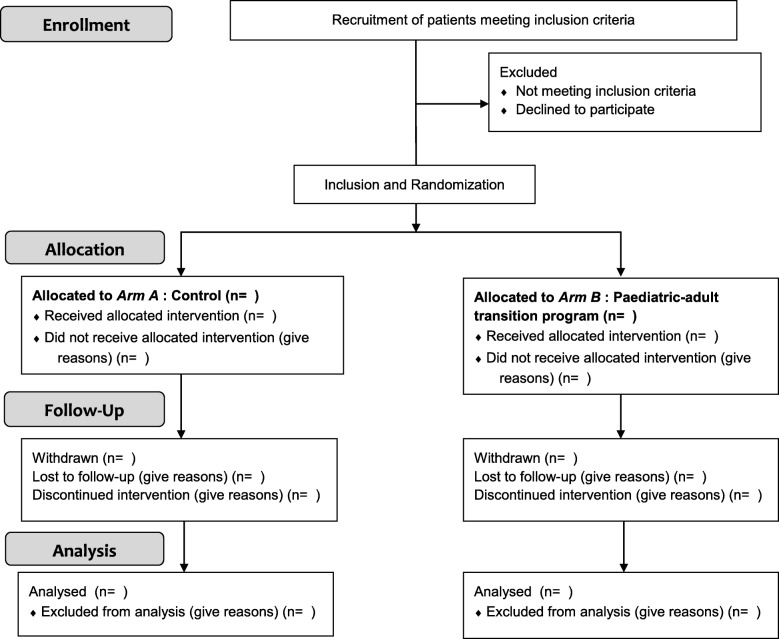
SPIRIT flow diagram of the DREPADO trial

**Fig. 2:**
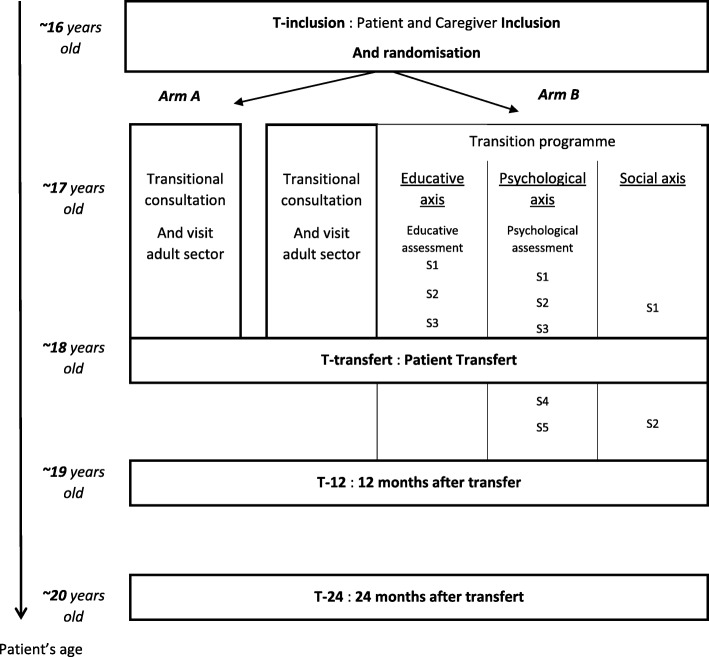
Detailed study scheme of the DREPADO trial

The Standard Protocol Items: Recommendation for Interventional Trials (SPIRIT) flow diagram is presented in Fig. 1 and the detailed study scheme in Fig. 2.

#### Arm A – control group

Patients and caregivers randomised to the control group receive the standard care management used in the study centre, including a joint consultation with a paediatrician and an internist/haematologist between the age of 16 and 18 years, and a visit to the adult department of care.

#### Arm B – intervention group

In addition to standard management, patients and caregivers randomised to the intervention group benefit from the SCD paediatric-adult transition programme. Based on the biopsychosocial approach, this consists of three structured axes, the objectives of which and practical details are summarised in Table 1.

**Table 1:** The three axes of paediatric-adult transition programme

Axes	Public	Objectives	Contents	Practical details	Providers
Educative	Patient and caregiver dyad	Providing self-care knowledge and skills related to SCD and therapeutics	Knowledge and coping skills, with patient needs assessment	Face to face At patients’ home 3 sessions of 2 h	One healthcare professional, trained in patient education and in SCD
Psychological	Individual patient i	Helping empower patients in relation to pain management and emotions	Knowledge and coping skills, with patient needs assessment	Face to face At hospital 6 sessions of 1.5 h	One healthcare professional, trained in therapeutic hypnosis and in SCD
Social	Group of 4 to 8 patients	Facilitating the relationship with oneself, others, and care	Coping skills	Face toface At ‘neutral place’ as association patient place 2 sessions of 2 h	One healthcare professional and one expert patient, both trained in patient education and in SCD

*h* hour*, SCD* sickle cell disease

The modifiable factors are used as coping skills during the educative, the psychological, and the social axis. The objective of the educative axis is to provide knowledge and coping skills related to self-care management. It is conducted at home with the patient and his/her parent by a healthcare professional trained in patient education. The objective of the psychological axis is to help empower patients in relation to pain management and emotions. It is conducted at hospital with only the patient by a healthcare professional trained in therapeutic hypnosis. The objective of the social axis is to facilitate the relationship to oneself, others, and care. It is conducted in a ‘neutral place’ (not at home and not at the hospital) with a group of patients by a healthcare professional trained in patient education and an expert adult patient also trained in patient education.

### Outcome measurements

The primary outcome is number of hospital admissions and emergencies in the index hospital for complications (i.e*.* VOC, ACS, and/or stroke) in the 2 years after T-transfer.

To evaluate the biopsychosocial approach, the secondary outcomes include bioclinical, psychological, and social factors, measured at T-inclusion, T-transfer, T-12, and/or T-24, collected from patients and/or parents. With regard to the indirect bioclinical factors, the use of care is evaluated by the incidence of emergency visits and medical consultations at T-24. Medication adherence is also measured at T-inclusion, T-transfer date, T-12, and T-24 by adapted Medication Intake Survey-Asthma (MIS-A) [17] adapted for SCD and Medication Adherence Report Scale (MARS) [18]. Psychological factors, such as pain perception, and social factors, such as school absenteeism is measured by self-declaration. Quality of life is evaluated using the brief World Health Organisation Quality of Life questionnaire (WHOQOL-Brief), and skills are measured by questionnaires, such as the health literacy using the European Health Literacy Questionnaire (HLS-EU-Q16) [19], self-efficacy feeling using the Self Efficacy Specific Instrument – Sickle Cell Disease (SCD-SES) [20]; these are measured at the T-inclusion, T-transfer, T-12, and T-24. All the data sources are presented in Table 2.

**Table 2:** Data collection plan

Outcomes		Time point				Population		Data sources
		T-inclusion	T-transfer	T-12	T-24	Patient	Parent	
Use of care	Hospitalisations for VOC, ACS, stroke		X		X	X		Medical records
	Emergency visits, medical consultations, imaging exams		X		X	X		Medical records
Medication adherence		X	X	X	X	X		2 questionnaires (MARS and adapted-MIS-A)
School absenteeism			X		X	X	X	Auto declaration
Quality of life		X	X		X	X		26-item questionnaire (WHOQOL-Brief)
Health literacy		X	X		X	X		16-item questionnaire (HLS-EU-Q16)
Disease and therapeutic knowledge		X	X		X	X	X	36-item questionnaire
Patient activation		X	X		X	X		13-item questionnaire
Sense of self-efficacy						X		9-item questionnaire (SCD-SES)
Transition preparation		X	X		X	X		20-item questionnaire (TRAQ)
Pain perceptions	At home			X	X	X		Auto declaration (book)
	Intensity during hospitalisations		X		X	X		Medical records

*ACS* acute chest syndrome, *HLS-EU-Q16* European Health Literacy Questionnaire, *MARS* Medication Adherence Report Scale, *adapted-MIS-A* Medication Intake Survey-Asthma, *SCD-SES* Self-Efficacy Specific instrument – Sickle Cell Disease, T*RAQ* Transition Readiness Assessment Questionnaire, *VOC* vaso-occlusive crisis, *WHOQOL-Brief* World Health Organization Quality of Life brief questionnaire

At the end of the study, the lived experience of a sample of patients, parents, and contributors is evaluated. The cost-effectiveness and the implementation of the transition programme are also secondary outcomes.

### Data collection and management

#### The data collection plan is presented in Table 2

The study data will be collected on a secure electronic case report form (eCRF) (Ennov Clinical, version 7.5.710.4, San Francisco, CA, USA), that will be available at each centre through an Internet portal. In each centre, eCRF users have their own personal login. A clinical research associate, delegated by the centre investigator and who is trained in GCP, enters data into the eCRF.

No personal identifying information will be mentioned on the eCRF. Each patient included the study will be assigned a unique identification number that will consist of the identification number of the investigational centre, the initials of the patient, and the chronological inclusion number of the patient.

Multiple external validation checks will be applied: examination of the source documents and cross-checking with the data recorded in the eCRF as to its accuracy, the presence of missing data, and the consistency of data. The eCRF will only include the data necessary for the analysis to be reported in a scientific publication.

All study data will be stored securely at Lyon University Hospital. All electronic data will be secured on a password-protected laptop. Paper-based study documents will be stored in a secure filing cabinet at each centre. All electronic documents containing names or personal identifying information, necessary for the follow-up of the study, will be stored separately from other study data and protected by a code number. Access to these files will be limited to research staff involved in the study.

The trial statisticians will have access to the data set for the final analysis of trial outcomes. They will receive checked and validated data from the eCRF with no personal identifying information.

### Monitoring and participant safety

The Trial Steering Committee (TSC) will be responsible for overseeing the progress of the trial and will meet at regular intervals. The TSC includes the principal investigator, the investigators of the centres, and the trial coordinators/project managers. The TSC has developed the study protocol and is responsible for data collection, management, publications, and the final data set. The committee is responsible for finding solutions to unforeseen questions/problems that may arise in the course of the study.

One monitoring session will be conducted per year in each centre by the clinical research department of the Lyon University Hospital. To ensure conformity to GCP, the medical records, informed consent forms, and the eCRFs will be checked.

No interim analysis or harms are expected, and therefore no premature stop of the trial is anticipated and no auditing by an independent committee is needed. According to French law, the study does not require a formal data monitoring committee as it is a trial with known minimal risks.

### Statistical analysis plan

Demographics and other characteristics are reported descriptively and according to treatment group. Means and standard deviation (SD), or medians and ranges when appropriate are calculated for continuous variables. Categorical variables will be presented using numbers and percentages.

The crude rate of hospitalisations due to complications will be calculated by dividing the total number of hospitalisations due to VOC, ACS, and stroke occurring after transfer by the total duration of follow-up of all patients in each group. The treatment effect will be assessed using a multivariable Poisson regression model with the study group as a factor, adjusting for minimisation factors and relevant patient characteristics and the length of follow-up as the offset. Results will be expressed as a rate ratio with 95% confidence interval. A negative binomial model will be fitted if data are over-dispersed. The same analyses will be performed for the association between intervention and other secondary outcomes.

Analysis of covariance (ANCOVA) will be used to evaluate the intervention effect at T-24 on all the secondary outcomes evaluated by questionnaires. Scores will be computed and depicted at each assessment by following the scoring procedures for each questionnaire. Each ANCOVA will include the baseline measure (T-inclusion) and minimisation factors as covariates. Changes over time for each score will be also assessed using generalized estimation equation analyses to test the effect of group, time, and the interaction group time after controlling for minimisation factors and relevant patient characteristics.

### Dissemination and data sharing statement

Important protocol modifications will be communicated to the relevant parties by sending the updated protocol to investigators.

There are no current plans for granting public access to the full protocol, participant-level data set or statistical code. However, if researchers wish to access the data set (e.g. to conduct a secondary analysis or meta-analysis) the project management committee will facilitate this.

The principal investigator will have access to the data and will take full responsibility for the analysis and publication of the results. Once the main analyses have been undertaken, data will be available upon reasonable request.

Results will be communicated through scientific publications, and one press release made in conjunction with a patient association (SOSglobi). Following this, an oral presentation will be given to the SCD health network (*Filière - MCGRE*) accredited by the French Ministry of Health.

## Discussion

Owing to the risk of discontinuity of care in chronic disease patients, it is essential to improve the paediatric-adult care transition. The DREPADO project is the first RCT designed to assess the impact of a paediatric-adult transition programme. The latter is based on the available literature [3] and on a recent previous qualitative study [10]. In accordance with this, it involves both paediatric and adult departments of care by starting the intervention in a paediatric department and continuing it in adult department of care. In addition, the qualitative study found that the needs of parents and patients were biopsychosocial, both individual and familial [10]. Added to that, the modifiable factors of the SMART [11, 12] were included in the three axes of the programme, to become patients’ coping skills. Furthermore, because the previous qualitative study [10] found the need for an integrated caregiver programme, the educative axis involves both the patient and their parent. This axis is conducted at home as this is patient’s environment as opposed to the hospital, which is the environment of care [21].

The primary outcome is bioclinical, which is more objective than complications measured in terms of pain. Secondary outcomes consider aspects related to the patient using a whole system approach, such as quality of life, but also include coping skills such as sense of self-efficacy, health literacy, and disease knowledge. In addition, as the transition of care is stressful and generally experienced by both patients and their parents, lived experiences of the proposed care transition programme are also evaluated.

The major strength of the DREPADO trial is the proposed intervention using a biopsychosocial, whole system approach to the patient, which aims to reflect as much as possible the real needs of patients and their family. In addition, needs are assessed at the beginning of the psychological and educative axes, in order to individualise the intervention. Another strength of the intervention is the participation of expert patients in the social axis; his/her experiential knowledge promotes sharing between the adolescents in the group and provides real-life examples of coping skills and advice [22]. The major limitation of DREPADO trial is the difficulty to implement the transition programme, as the intervention is complex [23]; it involves different departments of care, different places of intervention (home, hospital, ‘neutral place’), and different types of expertise (or the training of personnel in several different fields). This complexity requires good communication and coordination between interlocutors; to aid in this regard, the DREPADO study group provides each centre tools for communication and training. Another potential limitation is patient adherence to the programme, and the strategies to improve this are also based on communication, using, for example, paper schedules and reminder text messages.

The DREPADO RCT has multiple perspectives. First of all, for patients and their parents, this trial should show a better health status, quality of life, and a better experience of this difficult period of care. For healthcare professionals, it is expected to provide a model of paediatric-adult care transition for SCD. Furthermore, the methodological quality renders possible the evaluation of the efficacy and efficiency of the proposed programme. If conclusive, it will be possible to adapt it and test it in other chronic diseases presenting the same care transition problem [2]. For public health, the DREPADO trial results will be multiple. By focusing on this population of sub-Saharan origin with low visibility and high social vulnerability [24], this study will reduce the social inequalities in the healthcare system experienced by patients with SCD and their families. Also, by improving the health status, quality of life, and care of patients with SCD, the indirect cost of complications will decrease.

The DREPADO study is the first RCT designed to assess the impact of a paediatric-adult transition programme based on a biopsychosocial approach. By providing self-care knowledge and coping skills related to SCD and therapeutics, helping empower patients in relation to pain management and emotions, and facilitating the relationship to oneself, others, and care, we believe that the morbidity of patients with SCD may be reduced after the proposed transition programme.

## Supplementary information

**Additional file 1.**

**Additional file 2.** SPIRIT 2013 Checklist: Recommended items to address in a clinical trial protocol and related documents*.

## Data Availability

The data sets used and/or analysed during the current study available from the corresponding author on reasonable request.

## Abbreviations

ACS: Acute chest syndrome
eCRF: Electronic case report form
GCP: Good clinical practice
PREPS: Projet de Recherche sur la Performance du Système de Soins
RCT: Randomised controlled trial
SCD: Sickle cell disease
SMART: Social-Ecological Model of Adolescent and Young Adult Readiness to Transition
SPIRIT: Standard Protocol Items: Recommendation for Interventional Trials
T-12: 12 months after transfer
T-24: 24 months after transfer
T-inclusion: Baseline
T-transfer: Transfer date – which corresponds to the first consultation in the adult department of care
TSC: Trial Steering Committee
VOC: Vaso-occlusive crisis
WHOQOL: World Health Organisation Quality of Life Brief Questionnaire

## The DREPADO study group

contactable to etude.drepado@chu-lyon.fr is composed of: Delphine Hoegy, Audrey Janoly-Dumenil, Nathalie Bleyzac, Alexandra Gauthier-Vasserot, Giovanna Cannas, Arnaud Hot, Yves Bertrand, Pauline Occelli, Sandrine Touzet, Angélique Denis, Claude Dussart, Stéphanie Poupon-Bourdy, Fanny Nusbaum, Daniela Cuzzubbo, Karim Khadem, Ronald Guilloux.
