# Supplementary material for: Impact of a paediatric-adult care transition programme on the health status of patients with sickle cell disease: study protocol for a randomised controlled trial (the DREPADO trial)
Source: Trials. 2020 Feb 10;21:152. doi: 10.1186/s13063-019-4009-9 (PMC7008523; doi:10.1186/s13063-019-4009-9)
Supplement: Supplementary file 1 — Additional file 1. [file 13063_2019_4009_MOESM1_ESM.docx]

***
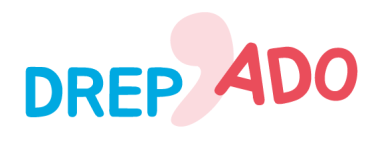
***

***Impact d’un programme de transition pédiatrie – adulte sur l’état de santé des adolescents atteints de drépanocytose : un essai contrôlé randomisé***

***Etude DREPADO***

***Version 3 du 26/10/2018***

**Promoteur** **:** Hospices Civils de Lyon

BP 2251

3 quai des Célestins,

69229 LYON cedex 02

**Investigateur coordonnateur :**

Pr Yves BERTRAND

Professeur d’Université, Praticien Hospitalier chef de service, hématologie

Pédiatrique

Institut d’Hématologie et d’Oncologie Pédiatrique – Hospices civils de Lyon

1, place Joseph Renaut – 69008 Lyon

Tel : 04 69 16 65 88

[yves.bertrand@ihope.fr](mailto:yves.bertrand@ihope.fr)

*Madame, Monsieur,*

*Le médecin de votre enfant ou un investigateur vous a présenté l’étude* ***DREPADO*** *et sollicite votre accord pour qu’il (elle) y participe.*

*Ce document a pour objectif de vous donner toutes les informations relatives à cette étude de façon à vous permettre d’exercer au mieux votre liberté de décision. Ce document est obligatoire et son contenu est défini par* [*le* ***Code de la Santé Publique, article L 1122-1***](http://www.legifrance.gouv.fr/WAspad/VisuArticleCode?commun=&code=&h0=CSANPUNL.rcv&h1=1&h3=11) *régissant les recherches impliquant la personne humaine.*

*Il décrit précisément l’étude et mentionne toutes les autorisations réglementaires obtenues pour sa mise en œuvre.*

*Avant de prendre une décision, il est important que vous lisiez attentivement ces pages qui vous apporteront les informations nécessaires concernant les différents aspects de cette recherche. Vous devez conserver ce document. N’hésitez pas à poser des questions si vous ne comprenez pas certains éléments.*

*La signature du formulaire de consentement devenue obligatoire par l'application du code de la Santé Publique (livre I, titres 2 et 3 du CSP), n'affecte aucunement vos droits légaux, ni ceux de votre enfant.*

*La participation de votre enfant est entièrement volontaire. Si vous ne désirez pas qu’il prenne part à cette recherche, ou si lui-même ne le souhaite pas, votre enfant continuera à bénéficier de la meilleure prise en charge médicale possible, conformément aux connaissances actuelles.*

**Pourquoi cette recherche?**

La drépanocytose entraîne des complications douloureuses, qui nécessitent une hospitalisation en urgence lorsqu’elles sont sévères. Ces complications peuvent être évitées par un suivi médical régulier et une prise des médicaments assidue depuis le plus jeune âge.

Aujourd’hui en France, c’est à l’adolescence que les patients atteints de drépanocytose ont le plus de complications. L’adolescence est une période de bouleversements psychologiques et d’adaptations des rôles familiaux et une période où le suivi médical passe du secteur enfant (la pédiatrie) au secteur adulte. Après le passage en secteur adulte, certains patients adolescents abandonnent leur suivi médical c’est-à-dire qu’ils manquent leurs rendez-vous médicaux, certains prennent aussi moins bien leurs médicaments. C’est pourquoi ils ont plus de complications.

Bien connaître la maladie et les médicaments, gérer différemment la douleur, arriver à s’orienter dans le système de soins permettraient d’améliorer le suivi médical en secteur adulte, de favoriser la bonne prise des médicaments et donc diminueraient le nombre de complications sévères.

Cette recherche permettra de confirmer cette idée.

**Quel est l’objectif de cette recherche?**

L’objectif de cette recherche est de diminuer le nombre de complications sévères liées à la drépanocytose, ayant conduits à une hospitalisation, dans les 24 mois suivant le transfert en secteur adulte.

Il y aura deux groupes de patients :

- Ceux qui auront un suivi classique,
- Ceux qui auront un suivi classique et en plus trois types d’interventions : sur la maladie et les traitements, sur la gestion de la douleur, sur l’orientation dans le système de soins.

Un tirage au sort est prévu pour savoir dans quel groupe votre enfant sera suivi.

**Quelle est la méthodologie de cette recherche ?**

Votre enfant atteint de drépanocytose SS, SC, Sβ sera inclus à partir de 16 ans (et jusqu’à 17 ans maximum)

Cette recherche durera jusqu’aux 21 ans environ de votre enfant.

Cette recherche nécessite 196 patients, répartis sur plusieurs centres en France.

**Comment va se dérouler cette recherche ?**

Si vous acceptez que votre enfant participe à cette recherche, un tirage au sort définira le groupe dans lequel sera suivi votre enfant pendant cette recherche :

-soit le groupe avec « suivi classique »: votre enfant bénéficiera alors d’un suivi habituel, c’est-à-dire une consultation avec le pédiatre tous les 6 mois, une consultation de transition avec le pédiatre et le médecin du secteur adulte après 16 ans, puis après son passage en secteur adulte une consultation avec le médecin du secteur adulte tous les 6 mois.

- soit le groupe avec « programme de transition » : votre enfant bénéficiera d’un suivi habituel à laquelle s’ajoutera un programme de transition défini de la façon suivante :

Entre les 16 et 19 ans de votre enfant, trois types d’interventions seront réalisées :

- **Intervention n°1** : Sur la maladie et les traitements :

3 séances maximum, d’environ 2h chacune

*Où ?* à domicile

*Qui ?* un intervenant, votre enfant et vous-même

- **Intervention n°2** : Sur la gestion de la douleur :

1 à 6 séances maximum, d’environ 1.5h chacune

*Où ?* avant ou après un rendez-vous avec le médecin, ou par téléphone

*Qui ?*  un intervenant et votre enfant uniquement

- **Intervention n°3**: Sur l’orientation dans le système de soins :

2 séances, d’environ 2h chacune

*Où ?* à l’hôpital ou à l’association de patients

*Qui ?* deux intervenants dont un représentant d’une association de patient, votre enfant et d’autres adolescents atteints de drépanocytose

Aucun bilan sanguin ne sera réalisé pour cette recherche.

Pendant ce suivi, nous recueillerons de manière confidentielle les données cliniques nécessaires à la recherche dans le dossier médical de votre enfant. Un cahier de suivi des douleurs vous sera remis, qui sera à compléter à domicile.

Dans les deux groupes, à certaines visites dans le centre pédiatrique ou adulte, nous demanderons à votre enfant et à vous-même de compléter des questionnaires sur diverses thématiques en lien avec la drépanocytose : la qualité de vie, la connaissance de la maladie, la prise des traitements, l’absentéisme scolaire ou la préparation à la transition pédiatrie-adulte.

Sur un échantillon de patients et de parents, à la fin de l’étude, un entretien pour discuter du vécu de la transition sera réalisé.

**Quels sont les bénéfices, les risques et les contraintes liés à la participation de votre enfant ?**

Les examens, prélèvements pour bilan sanguin, et médicaments seront similaires s’il est inclus dans la recherche ou s’il n’est pas inclus.

Pour le groupe « programme de transition », les bénéfices:

- sont directs pour votre enfant par un suivi plus rapproché et prenant en compte ses besoins.

- pour vous-même sont liées à votre implication dans le suivi de votre enfant adolescent.

Les contraintes sont :

- liées à l’évaluation, pour le groupe avec « suivi classique » et le groupe « programme de transition » :

Bien que non souhaitée, le remplissage des questionnaires peut être vécu comme contraignant pour vous-même et votre enfant. Il nécessite une certaine disponibilité lors des venues à l’hôpital.

- liées aux interventions, pour le groupe « programme de transition » :

Bien que non souhaitée, la venue d’un professionnel de santé à domicile pour l’intervention n°1 sur la maladie et les traitements peut être mal vécue par vous-même ou votre enfant.

La venue à l’hôpital ou à l’association de patients pour l’intervention n°3 sur l’orientation dans le système de soins entraîne une contrainte de transports.

**Quels sont vos droits ?**

***Volontariat***

Vous êtes entièrement libre d’accepter ou de refuser que votre enfant participe à cette recherche, sans que cela ne modifie la qualité des soins ou les relations existant avec son médecin.

Pour pouvoir participer à cette étude, votre enfant doit nécessairement être affilié (par votre intermédiaire) à un régime d’assurance maladie telle que celui de la sécurité sociale.

**Si vous changez d’avis pendant la recherche :**

Vous pouvez à tout moment demander à interrompre la participation de votre enfant à l’étude, sans justification de votre part. Dans ce cas, les données de votre enfant recueillies jusque-là seront utilisées dans les résultats de l’étude.

Si le médecin juge nécessaire pour le bien de votre enfant, il pourra modifier son suivi. Votre enfant continuera à bénéficier pleinement de sa compétence.

**Si vous souhaitez des informations :**

A tout moment, toutes les informations que vous souhaitez obtenir concernant cette recherche vous seront communiquées, dans la mesure du possible par le médecin de votre enfant.

Vous serez tenus informés de toute nouvelle donnée importante concernant l’étude à laquelle vous acceptez que votre enfant participe.

**Réutilisation des données :**

Sauf opposition expresse de votre part adressée au médecin, les données de votre enfant recueillies dans le cadre de cette étude pourront être transmises ailleurs dans le monde et réutilisées par des partenaires publics ou privés lors de recherches ultérieures exclusivement réservées à des fins scientifiques.

Si vous avez des questions ou des réclamations au sujet du traitement des données de votre enfant au cours de cette étude, vous pouvez contacter le DPO par voie électronique : dpo@chu-lyon.fr ou par courrier postal :

Le délégué à la protection des données

162 avenue Lacassagne

Bâtiment A – 3e étage – Bureau 316

69003 LYON

Si vous estimez, après avoir contacté le DPO des HCL, que vos droits sur les données de votre proche ne sont pas respectés, vous pouvez adresser une réclamation (plainte) à la CNIL :

https://www.cnil.fr/fr/webform/adresser-une-plainte

**Interruption de la recherche par le médecin :**

S’il juge que c’est dans l’intérêt de votre enfant, le médecin tout comme le promoteur peut interrompre à tout moment la participation de votre enfant à la recherche, ou arrêter l’étude pour des raisons médicales, administratives ou autres.

**Participation à d’autres recherches :**

La participation de votre enfant à cette étude n’empêche pas la participation de votre enfant à une autre recherche.

**Coût/Rémunération :**

Il n’y aura pas de coût supplémentaire pour vous.

Ni vous, ni votre enfant ne serez rémunérés du fait de la participation à l’étude.

**Une fois que votre enfant sera devenu majeur, nous solliciterons son accord écrit pour la poursuite de sa participation à l’étude DREPADO.**

**Confidentialités et protection des données**

Dans le cadre de cette recherche interventionnelle à risques et contraintes minimes, un traitement informatique des données personnelles de votre enfant va être mis en œuvre pour permettre d’analyser les résultats de la recherche. Le responsable du traitement des données est le promoteur, dont les coordonnées figurent sur la première page de ce document. Ce traitement des données a pour fondement juridique l’article 6 du Règlement Général sur la Protection des Données (RGPD) à savoir l’exécution d’une mission d’intérêt public dont est investi le responsable de traitement et les intérêts légitimes poursuivis par lui. De plus, au titre de l’article 9 du RGPD le responsable de traitement peut de manière exceptionnelle traiter des catégories particulières de données, incluant des données de santé notamment à des fins de recherche scientifique.

Pour l’analyse, les données médicales concernant votre enfant seront transmises aux Hospices Civils de Lyon ou aux personnes agissant pour son compte en France. En cas de transfert de données à caractère personnel hors de l’Union Européenne et/ ou vers un pays ne garantissant pas un niveau de protection suffisant par rapport à l’Union Européenne ou à une organisation internationale, le promoteur et/ou le responsable de traitement mettront en place des garanties appropriées pour ce transfert (Clauses Contractuelles Spécifiques). Si vous souhaitez obtenir une copie des Clauses Contractuelles Spécifiques, vous pouvez vous adresser au Délégué à la Protection des Données (DPO) du promoteur à l’adresse suivante : dpo@chu-lyon.fr. Ces données seront identifiées par un code et ses initiales. Ces données pourront également, dans des conditions assurant leur confidentialité, être transmises aux autorités de santé françaises et à d’autres entités en dehors des Hospices Civils de Lyon. Les données seront transférées et collectées conformément à la méthodologie de référence MR001 de la Commission Nationale de l’Informatique et des Libertés (CNIL) pour laquelle les Hospices Civils de Lyon ont signé un engagement de conformité. Conformément à la réglementation française et européenne, les données de l’étude seront conservées 25 ans.

Vous pourrez également, à tout moment, exercer votre droit d’accès, de vérification, de correction , de limitation et d’opposition au traitement et à la transmission des données concernant votre enfant en faisant la demande auprès du médecin. Si vous souhaitez exercer votre droit à l’effacement des données de votre proche, le responsable de traitement peut au titre des Articles 17.3.c et 17.3.d. du RGPD ne pas faire droit à cette demande si celle-ci est susceptible de rendre impossible ou de compromettre gravement la réalisation des objectifs de la recherche. Ainsi, les données de votre proche recueillies préalablement au retrait de votre consentement pourront ne pas être effacées et pourront continuer à être traitées dans les conditions prévues par la recherche.

Si les résultats de cette étude devaient être présentés dans des communications et des publications scientifiques médicales, l’identité des participants n’apparaîtra d’aucune façon.

A l’issue de l’étude, les résultats globaux de la recherche pourront vous être communiqués sur simple demande auprès du médecin de votre enfant. La base de données de l’étude rendue totalement anonyme pourra être transmise à d’autres chercheurs qui travailleraient sur le même sujet.

**Dispositions réglementaires**

Le Comité de Protection des Personnes Sud-Ouest et Outre-Mer III (Groupement Hospitalier Pellegrin – Place Amélie Raba Léon – 33076 Bordeaux Cedex) a émis un avis favorable à la réalisation de cette étude le 26/09/2018. Enfin, cette recherche respecte le règlement sur la protection des données.

Le promoteur de cette recherche, les Hospices Civils de Lyon, BP 2251, quai des célestins, 69229 Lyon cedex 02, a souscrit une assurance de responsabilité civile auprès de la Société Hospitalière d’Assurance Mutuelle, 18 rue Edouard Rochet, 69008 Lyon, sous le numéro 153.930.

Les personnes ayant subi un préjudice après participation à une recherche interventionnelle *à risques et contrainte minimes* peuvent faire valoir leurs droits auprès de l’assureur promoteur.

L’investigateur doit vous fournir toutes les explications nécessaires concernant cette recherche. Si vous souhaitez en retirer votre enfant à quelque moment que ce soit, et quel que soit le motif, votre enfant continuera à bénéficier du suivi médical et cela n'affectera en rien sa surveillance future.

**Qui pouvez-vous contacter pour toute question ?**

Si vous avez des questions concernant l’étude, n’hésitez pas à nous les poser. Nous pouvons vous donner les informations complémentaires que vous souhaitez.

Les noms et numéros de téléphone des personnes à contacter sont les suivants :

Investigateur coordonnateur de l’étude

Pr Yves BERTRAND

Service Hématologie Pédiatrique

Institut d’Hématologie et d’Oncologie Pédiatrique (IHOPe)/ Hospices Civils de Lyon - Groupement Est

1 place J. Renaut - 69008 Lyon

Tél : 04.69.16.65.50

Chef de projet de l’étude

Dr Delphine HOEGY

Pharmacie

Hôpital Edouard Herriot/ Hospices Civils de Lyon

5 place d’Arsonval - 69003 Lyon

Tél : 04.72.11.09.98

[etude.drepado@chu-lyon.fr](mailto:etude.drepado@chu-lyon.fr)

Investigateur de votre centre référent pour l’étude (selon les centres)

Titre Prénom NOM

Service

Hôpital/Groupement

Adresse

Tél : XX.XX.XX.XX.XX

Nous vous remercions de l’attention que vous avez portée à la lecture de cette notice. Une copie de ce document vous sera remise pour que vous puissiez bénéficier de l’ensemble des informations concernant la participation de votre enfant à l’étude.

Lorsque vous aurez lu cette note d’information, il vous sera proposé, si vous êtes d’accord, de donner votre consentement écrit en signant le formulaire préparé à cet effet.

| 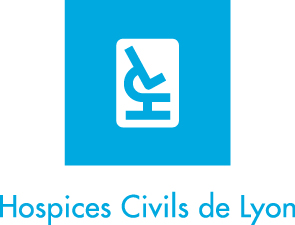  **Direction de la Recherche Clinique**  **et de l’Innovation** | **FORMULAIRE DE CONSENTEMENT A L’ATTENTION**  **DES PARENTS** |
| --- | --- |

**La loi 2012-300 du 5 mars 2012 relative aux recherches impliquant la personne humaine rend obligatoire le recueil de l'accord écrit des parents des enfants sollicités pour participer à toute recherche interventionnelle ou recherche interventionnelle à risques et contraintes minimes. C'est un tel accord qui vous est demandé ci-dessous, pour que votre enfant participe à l’étude intitulée :**

***
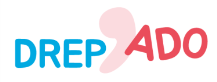
***

***Impact d’un programme de transition pédiatrie – adulte sur l’état de santé des adolescents atteints de drépanocytose : un essai contrôlé randomisé***

***Etude DREPADO***

**Promoteur** **:** Hospices Civils de Lyon

BP 2251

3 quai des Célestins,

69229 LYON cedex 02

**Investigateur coordonnateur :**

Pr Yves BERTRAND

Professeur d’Université, Praticien Hospitalier chef de service, hématologie

pédiatrique, responsable médical

Institut d’Hématologie et d’Oncologie Pédiatrique – Hospices civils de Lyon

1, place Joseph Renaut – 69008 Lyon

Tel : 04 69 16 65 88

[yves.bertrand@ihope.fr](mailto:yves.bertrand@ihope.fr)

Je soussigné, …………………………………………………………………………………………… *(NOM, Prénom)*, parent ou tuteur légal de …………………………..…………………..……………….…… *(NOM, Prénom)* né(e) le ….……..………….…….., *(JJ/MM/AAAA)* certifie avoir lu et compris la note d’information qui nous a été remise.

J’ai eu la possibilité de poser toutes les questions que je souhaitais au Pr/Dr*/Mr/Mme (nom, prénom)* qui m’a expliqué la nature, les objectifs, les risques potentiels et les contraintes liées à ma participation à cette recherche. Je connais la possibilité qui m’est réservée d’interrompre la participation de mon enfant à cette recherche, à tout moment sans avoir à justifier cette décision et je ferai mon possible pour en informer le médecin. Cela ne remettra naturellement pas en cause la qualité des soins de mon enfant.

J’ai eu l’assurance que les décisions qui s’imposent pour la santé de mon enfant seront prises à tout moment, conformément à l’état actuel des connaissances médicales.

J’ai bien compris que le médecin peut interrompre à tout moment la participation de mon enfant à la recherche, s’il le juge nécessaire.

Je suis informé(e) de la possibilité que les données recueillies dans cette recherche puissent être réutilisées lors de recherches ultérieures exclusivement à des fins scientifiques et que nous pouvons nous y opposer.

J’ai été informé(e) que cette recherche a reçu l’avis favorable du Comité de Protection des Personnes Sud-Ouest et Outre-Mer III le 26/09/2018 et a fait l’objet d’une déclaration à la Commission Nationale Informatique et Libertés (CNIL). J’ai bien noté que cette recherche est menée conformément aux articles L1121-1 et suivants du Code de la Santé Publique, relatifs à la protection des personnes qui se prêtent à des recherches impliquant la personne humaine et conformément à la règlementation en vigueur.

Je certifie sur l’honneur que mon enfant est affilié à un régime de sécurité sociale ou bénéficiaire d’un tel régime.

Le promoteur de la recherche, les Hospices civils de Lyon, BP 2251, quai des célestins, 69229 Lyon cedex 02 a souscrit une assurance de responsabilité civile en cas de préjudice auprès de de la Société Hospitalière d’Assurance Mutuelle, 18 rue Edouard Rochet, 69008 Lyon, sous le numéro 153.930.

J’accepte que les personnes qui collaborent à cette recherche ou qui sont mandatées par le promoteur, ainsi qu’éventuellement le représentant des Autorités de Santé, aient accès à l’information contenue dans le dossier médical de notre enfant dans le respect le plus strict de la confidentialité. J’accepte que les données enregistrées à l’occasion de cette recherche, puissent faire l’objet d’un traitement informatisé sous la responsabilité du promoteur.

J’ai bien noté que, conformément aux dispositions de la loi relative à l’informatique, aux fichiers et aux libertés, nous disposons d’un droit d’accès, de rectification, de vérification et de correction des données. Je dispose également d’un droit d’opposition à la transmission des données couvertes par le secret professionnel susceptibles d’être utilisées dans le cadre de cette recherche et d’être traitées. Ces droits s’exercent auprès du médecin qui suit mon enfant dans le cadre de cette recherche et qui connaît notre identité.

Mon consentement ne décharge en rien l’investigateur et le promoteur de la recherche de leurs responsabilités à l’égard de mon enfant. Mon enfant et moi-même conservons tous les droits garantis par la loi.

Si nous en faisons la demande, les résultats globaux de la recherche seront communiqués directement, conformément à la loi du 4 mars 2002 relative aux droits des malades et à la qualité du système de santé.

Je peux à tout moment demander des informations complémentaires au  Pr/Dr à Mr/Mme ………………………………………….. *(nom, prénom).* *(à modifier selon les centres)*

Deux exemplaires originaux de ce formulaire de consentement ont été établis : un nous a été remis, l’autre a été remis à l’investigateur et sera conservé au minimum 25 ans après la fin de la recherche.

## Parents donnant leur consentement :

## Ayant disposé d’un temps de réflexion suffisant avant de prendre notre décision, nous acceptons librement et volontairement :

## que notre enfant …………………………………..…………… *(NOM, Prénom)* participe au projet de recherche DREPADO.

| NOM, Prénom du 1^er^ titulaire de l’autorité parentale :  …………………………………………..………..  Fait à ……………………………………..  Le : \|__\|__\|/\|__\|__\|/\|__\|__\|__\|__\|  Signature : | NOM, Prénom du 2ème titulaire de l’autorité parentale (non obligatoire) :  …………………………………………..………..  Fait à ……………………………………..  Le : \|__\|__\|/\|__\|__\|/\|__\|__\|__\|__\|  Signature : | NOM, Prénom du patient si celui-ci veut apposer sa signature :  …………………………………………..………  Fait à ……………………………………..  Le : \|__\|__\|/\|__\|__\|/\|__\|__\|__\|__\|  Signature : |
| --- | --- | --- |

## Investigateur obtenant le consentement :

## J’atteste que toutes les obligations liées à un consentement éclairé ont été satisfaites dans le cadre de ce projet de recherche clinique – que les parents du participant ont reçu une information relative à leur droits, que nous avons discuté de ce projet et que je leur ai expliqué en termes compréhensibles l’ensemble des informations contenues dans la notice. Je certifie également avoir laissé les parents du participant me poser toutes les questions qu’ils souhaitaient et y avoir répondu.

NOM, Prénom de l’investigateur : ……………………………………………………

Fait à : …………………………………., le |__|__|/|__|__|/|__|__|__|__|

Signature de l’investigateur :
